# Elements in Invasive Redclaw Crayfish *Cherax quadricarinatus* Pose Human Health Risks in the Largest Floodplain System of South Africa

**DOI:** 10.1007/s00128-024-03963-1

**Published:** 2024-10-11

**Authors:** Johannes H. Erasmus, Wynand Malherbe, Nico J. Smit, Victor Wepener

**Affiliations:** Water Research Group, Unit for Environmental Sciences and Management, North-West University, Potchefstroom, 2520 South Africa

**Keywords:** Phongolo River Floodplain, Conservation area, Metal bioaccumulation, Australian redclaw crayfish, Carcinogenic and non-carcinogenic risk

## Abstract

The Australian redclaw crayfish, *Cherax quadricarinatus*, has been introduced globally for aquacultural purposes, and invasive wild populations have established in several countries. An increase in population growth, has caused several low-income and rural communities to utilise it as an inexpensive protein source. Despite this introduction, limited research has been done on element accumulation, and the risks for human consumption. This study focused on elemental accumulation in *C. quadricarinatus* from two rivers in the Phongolo River Floodplain (PRF), South Africa. The non-carcinogenic and carcinogenic human health risks associated with its consumption were calculated. The accumulation order in the PRF was Zn > Cu > Pb > Cr > As > Ni > Hg > Cd. Elevated concentrations of As and Hg, as well as As, Cr, and Ni posed non-carcinogenic and carcinogenic risks, respectively. These results highlight that wild populations of *C. quadricarinatus* not only pose a threat to aquatic ecosystems but also potentially cause human health risks when consumed.

**Supplementary Information:**

The online version contains supplementary material available at 10.1007/s00128-024-03963-1.

## Introduction

The Australian redclaw crayfish, *Cherax quadricarinatus*, has been introduced on a global scale due to their aquaculture potential and aquarium trade purposes (Nunes et al. 2017a). This crayfish species has established invasive wild populations in several southern African countries, including Eswatini, Mozambique, South Africa, Zambia, and Zimbabwe (Madzivanzira et al. 2020, 2023). With an increase in wild populations, several low-income and rural communities are increasingly utilising them as an easily accessible and inexpensive protein source (Nunes et al. 2017b). These crayfish are in close contact with sediments and are known to accumulate and concentrate trace elements in their body tissues (Nakayama et al. 2010). This can pose potential health risks to local consumers if they are chronically exposed to elevated trace element concentrations in crayfish tissues. Local communities that might be at risk are those living in the Phongolo River Floodplain (PRF). The PRF is considered the most important floodplain system in South Africa, due to its high ecological and social-economic role (Dube et al. 2017; Volschenk et al. 2019).

The PRF is South Africa’s largest floodplain system covering an area of 13,000 ha during full inundation and is influenced by two major rivers, the Phongolo and uSuthu rivers (Acosta et al. 2021). These two major rivers drain large-scale agricultural activities, especially sugar cane, while in the upper catchments several other anthropogenic activities, such as industrial, forestry and mining activities also occur (van Rooyen et al. 2022). According to literature, the intensity of these activities is lower in the uSuthu River, compared to the Phongolo River (Hendriks and Rossouw 2009; IUCMA 2014; Vilane and Tembe 2016; de Necker et al. 2019; van Rooyen et al. 2022). The confluence of these two major rivers occurs in the Ndumo Game Reserve (NGR), the only protected area within the PRF (de Necker et al. 2019). The first report of wild populations of *C. quadricarinatus* in the NGR was in 2012 (du Preez and Smit 2013) and only three years later, it was already established that local communities are utilising this invasive crayfish. A study on fish consumption preferences conducted in the Ndumo area, revealed that 21% of the respondents consume *C. quadricarinatus* with a mean frequency of 2.3 individual crayfish per week (Coetzee et al. 2015). The local fishing community not only actively fish for this crayfish (32% of fishers caught this species, with a mean of 6.9 crayfish caught per week), but they also sell them to the community: with a mean of 13.9 crayfish per week (Coetzee et al. 2015).

Several studies have assessed the ecological and invasive impacts of *C. quadricarinatus* on southern African river systems (de Moor 2002; Nunes et al. 2017b; South et al. 2019, 2020; Madzivanzira et al. 2021, 2022; Zengeya et al. 2022), however, only one study in southern Africa assessed element bioaccumulation in these crayfish (Nakayama et al. 2010), and no information is available on the risks that consumption may pose to humans. Thus, the present study aimed to assess the element accumulation in *C. quadricarinatus* from the PRF and the potential non-carcinogenic and carcinogenic human health risks it may pose.

### Materials and Methods

#### Field Sampling

**Fig. 1:**
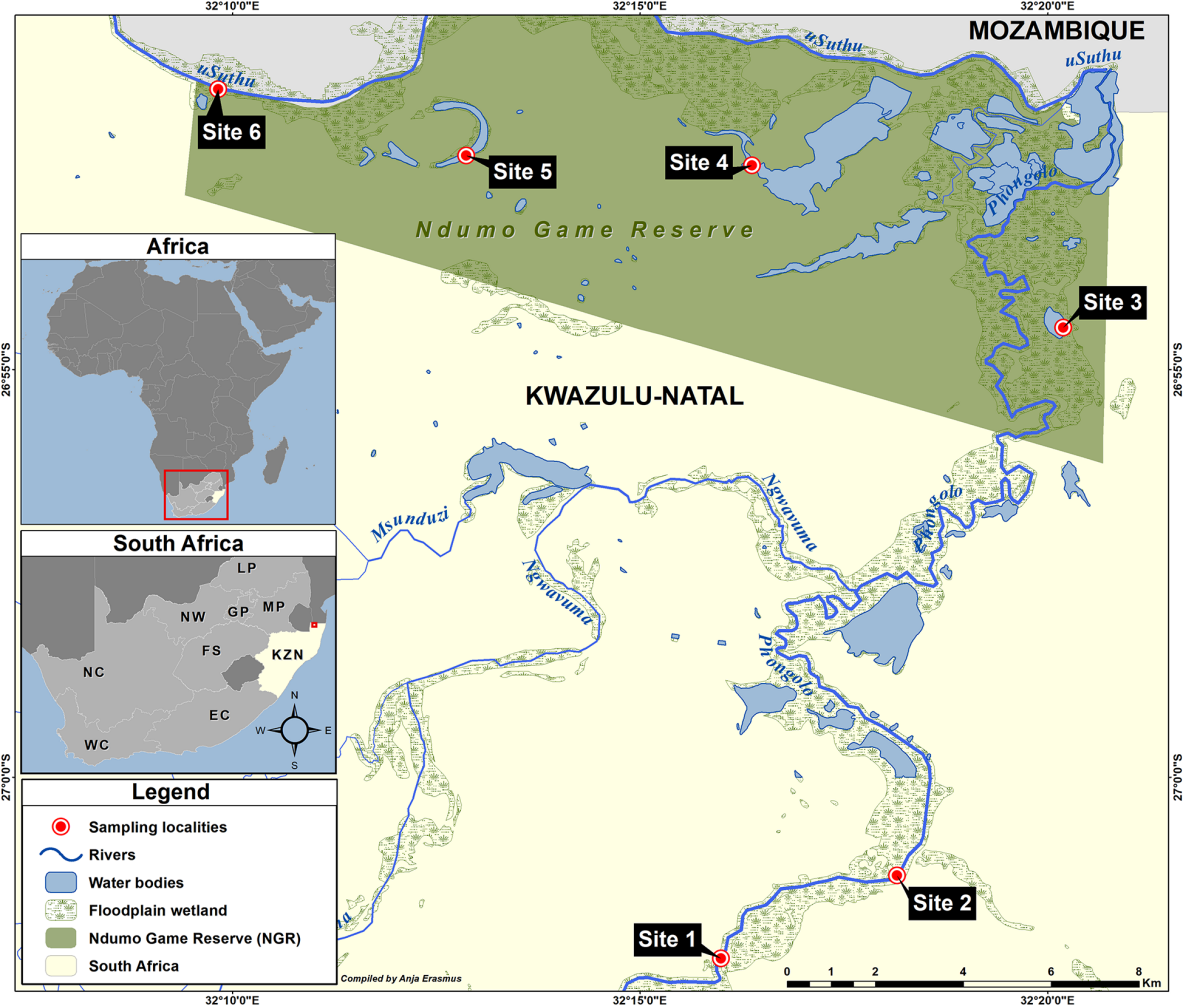
Map of the study area located in the Phongolo River Floodplain, South Africa. Sites 1–3 are associated with the Phongolo River, while Sites 4–6 are associated with the uSuthu River

Sampling was conducted at three sites that are associated with the Phongolo River and three sites that are associated with the uSuthu River (Fig. 1). A total of 32 *C. quadricarinatus* individuals were collected from the six sites in and around the NGR during September 2022 (Supplementary data, Table S1). Sites 1 and 2 were in the Phongolo River outside of the NGR, while Site 3 was in a floodplain lake that receives water from the Phongolo River. Sites 4 and 5 were in two floodplain lakes that receive water from the uSuthu River, while Site 6 was in the uSuthu River. The necessary ethics and permit were obtained prior to sampling (NWU-00156-18-A5; OP 2792/2022). *Cherax quadricarinatus* were caught by using an electrofisher (LR-24, Smith-Root), each individual was weighed and measured (total body length) and subsequently euthanised and dissected. The muscle tissue from the tail was collected in pre-cleaned polypropylene tubes and frozen until further analysis.

#### Laboratory Analyses

Crayfish tail muscle tissues were freeze-dried (FreeZone 6, Labconco), whereafter approximately 0.2 g dry tail tissue was acid digested (Ethos Easy, Milestone) in a mixture of 7.5 ml HNO_3_ (65%, supra pure quality, Merck) and 2.5 ml HCl (37%, supra pure quality, Merck) as described in Erasmus et al. (2023). Digested samples were transferred to 50 ml volumetric flasks and diluted with 1% HNO_3_ and, subsequently transferred to pre-cleaned polypropylene tubes until elemental analyses. Samples were analysed for multi-element concentrations (As, Cd, Cu, Ni, Pb and Zn) using inductively coupled plasma mass spectroscopy (ICP-MS) (7700 series, Agilent), whereas Cr and Hg were analysed using graphite furnace atomic absorption spectroscopy (GF-AAS) (PinAAcle 900T, PerkinElmer) and a flow injection mercury system (FIMS) (FIMS 400, PerkinElmer), respectively, due to interferences. Instrument operational settings, calibration methods, quality assurance and sample preparation were as described in Erasmus et al. (2020) for ICP-MS and GF-AAS, as well as in Erasmus et al. (2022a) for FIMS analyses. Quality control of element concentrations in biological tissue was performed using certified reference material (DORM-5, fish protein, National Research Council Canada) and the recovery rates for all eight elements were within 20% of the certified concentrations (Table 1).

**Table 1:** Limit of detection (LOD) and limit of quantification (LOQ) reported in µg/g dry weight (DW) in *Cherax quadricarinatus* samples, as well as the recovery rates (%) from certified reference material for the elements of interest

Elements	LOD (µg/g DW)	LOQ (µg/g DW)	DORM-5 (% recovery)
As	0.0011	0.0032	92
Cd	0.00028	0.00085	114
Cr	0.0066	0.0199	113
Cu	0.056	0.168	88
Hg	0.00024	0.00071	108
Ni	0.0048	0.0145	106
Pb	0.0013	0.0040	88
Zn	0.029	0.088	116

### Statistical Analyses

Data were tested for homogeneity of variance and normality using Shapiro-Wilk normality test, whereafter one-way ANOVAs with Tukey’s multiple comparison tests were used to test for significant differences (*p* < 0.05) in element concentrations, as well as human health risks between the sites, using GraphPad Prism v10.2.3. A redundancy analysis (RDA) was constructed to assess the bioaccumulation patterns in crayfish tail tissues and relate it to the corresponding biometric data of crayfish from the two major rivers and their associated floodplain lakes using Canoco v5.15. All the data used for the RDA was log transformed y = log (x + 1). The human health risk assessment was performed to assess the non-carcinogenic and carcinogenic health risks associated with the consumption of *C. quadricarinatus*, as well as the maximum safe consumption limits from sites in the PRF. These calculations were based on the equations and guidelines described in Erasmus et al. (2022b).

## Results

### Element Bioaccumulation in *Cherax quadricarinatus*

**Fig. 2:**
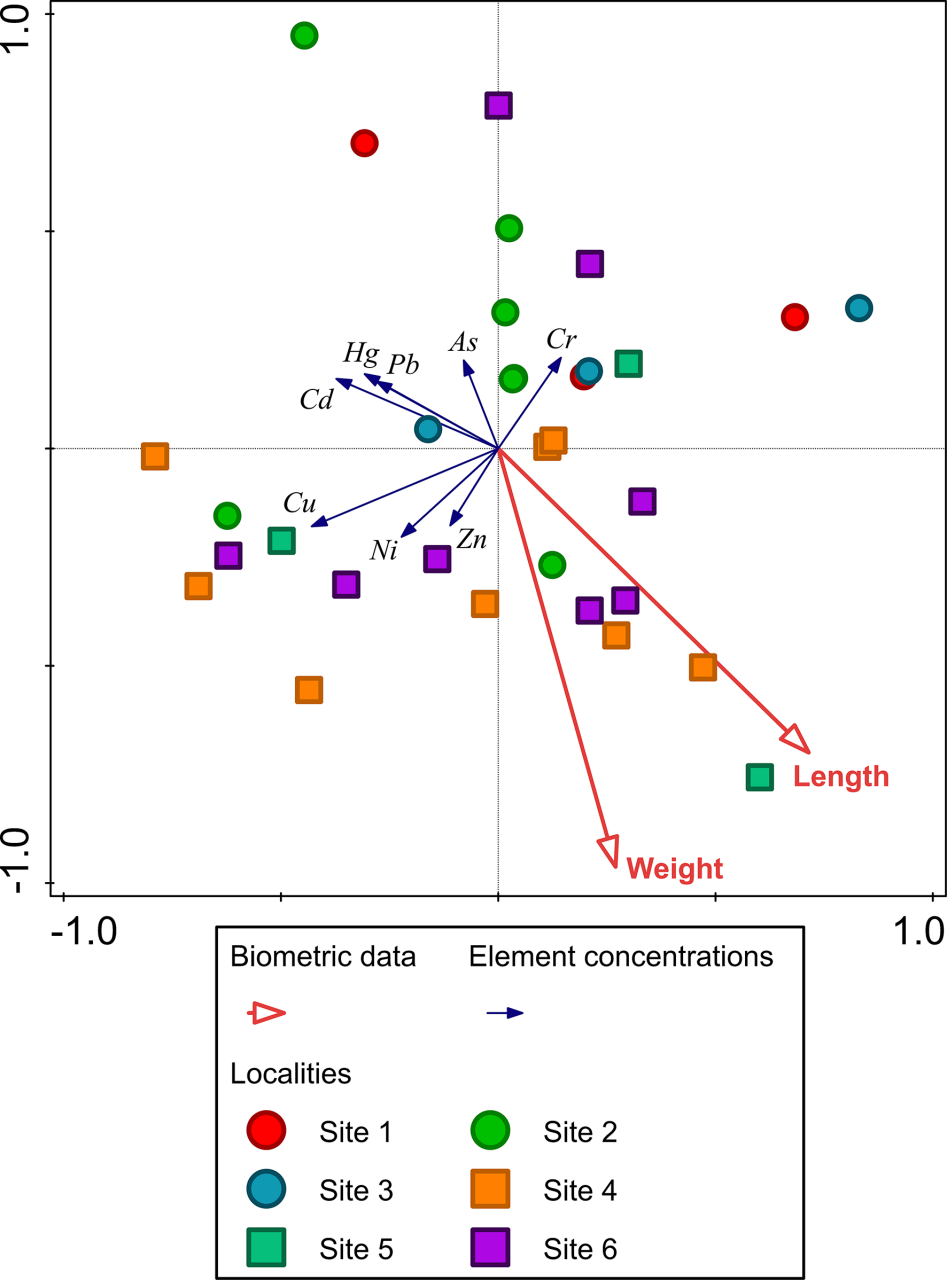
RDA triplot illustrating the associations between element concentrations and biometric variables in *Cherax quadricarinatus* collected from sites in the Phongolo (circles) and uSuthu (squares) river basins. The triplot explains 36.3% of the variation, with 25.6% on the first axis and 10.7% on the second axis

All eight element concentrations in *C. quadricarinatus* were generally higher in the Phongolo River sites compared to its associated lake site, while concentrations of Cd, Cu, Pb and Zn were higher in the uSuthu River site compared to its associated lake sites (Supplementary data, Figures S1 and S2). *Cherax quadricarinatus* collected from the sites associated with the uSuthu River (Sites 4–6), were in general larger and heavier individuals with more individuals (Fig. 2; Supplementary data Table S1). These individuals also associated with higher concentrations of essential elements (Cu, Ni and Zn) compared to the individuals collected from sites associated with the Phongolo River (Sites 1–3) (Fig. 2). *Cherax quadricarinatus* collected from the sites associated with the Phongolo River, generally had higher non-essential element concentrations (As, Cd, Cr, Hg, Pb), where Site 2 had significantly higher concentrations of As compared to Site 4 (*p* = 0.0003) and Site 6 (*p* = 0.0017), as well as Cr compared to Site 6 (*p* = 0.0240), respectively (Fig. 2; Supplementary data, Figures S1 and S2).

### Human Health Risks

To date, most human health risk assessments are based on a generalised assumption that a person of 60 kg consuming a fish or crayfish meal (150 g) twice a week (Heath et al. 2004; Erasmus et al. 2022b). However, for this study we used the actual consumption values for this region as determined by Coetzee et al. (2015) together with our own data on the weight of the crayfish to calculate the realistic risk. Therefore, the calculations were based on a person of 60 kg consuming a crayfish meal of 105 g 2.3 times a week for non-carcinogenic risks and daily for carcinogenic risks. For the non-carcinogenic risks, a hazard quotient (HQ) greater than one indicates a high probability of adverse health effects, while for the carcinogenic risks, a cancer risk (CR) above 10^− 4^ is considered unacceptable (Erasmus et al. 2022b). Of the eight elements assessed, only As and Hg posed non-carcinogenic risks, while As, Cr and Ni posed carcinogenic risks (Table 2). Concentrations of As at Sites 1 and 2 had a HQ > 1, while the concentrations of Hg exceeded the HQ at all sites and ranged between 1.3 and 1.6 (Table 2). Unacceptable CR associated with As and Cr were observed at all the sites, as well as Ni at all the sites, except Site 3, and ranged between 5.6 and 13.5 for As, 1.4 and 7.2 for Cr, and 0.82 and 4.0 for Ni (Table 2). *Cherax quadricarinatus* at Site 2 posed significantly higher non-carcinogenic and carcinogenic risks for As compared to Site 4 (*p* = 0.0005; *p* = 0.0004) and Site 6 (*p* = 0.0028; *p* = 0.0022), as well as carcinogenic risks for Cr compared to Site 6 (*p* = 0.0236).

**Table 2:** The mean and standard deviation of hazard quotients (HQs) for non-carcinogenic risk, cancer risk (CR) and maximum safe consumption limit per day for *Cherax quadricarinatus* collected from the Phongolo River Floodplain, South Africa. The values were calculated on the element concentration in crayfish tail tissue supposing a person of 60 kg consumes one crayfish meal (105 g) 2.3 times a week for the HQ value, or daily for the CR value. Hazard quotients and CR values for most of the investigated elements were far below zero (see supplementary data, table S2) and only values of HQ > 1, indicating a high probability of adverse health effects, and CR > 10^− 4^, indicating an unacceptable risk to humans who consume these crayfish (in bold), are included in this table

	Phongolo River			uSuthu River		
Elements	Site 1	Site 2	Site 3	Site 4	Site 5	Site 6
Hazard quotient for non-carcinogenic risk (HQ)						
As	**1.0 ± 0.12**	**1.3 ± 0.27**	0.82 ± 0.19	0.51 ± 0.10	0.86 ± 0.27	0.61 ± 0.42
Hg	**1.5 ± 0.39**	**1.6 ± 0.42**	**1.2 ± 0.19**	**1.4 ± 0.27**	**1.6 ± 0.07**	**1.3 ± 0.48**
Carcinogenic risk (CR) (10^− 4^)						
As	**11.0 ± 1.3**	**13.5 ± 3.0**	**8.9 ± 2.1**	**5.6 ± 1.0**	**9.3 ± 2.9**	**6.6 ± 4.5**
Cr	**7.2 ± 6.4**	**6.4 ± 3.5**	**1.4 ± 0.97**	**3.3 ± 1.6**	**1.6 ± 0.04**	**1.8 ± 0.58**
Ni	**2.8 ± 1.3**	**2.1 ± 2.6**	0.82 ± 0.19	**4.0 ± 5.8**	**1.1 ± 0.27**	**2.1 ± 1.0**
Maximum safe consumption limit per day (g)						
As (NC)	43.5 ± 5.4	36.8 ± 9.0	55.0 ± 11.3	87.5 ± 15.2	54.2 ± 16.3	86.4 ± 25.0
As (CR)	217.4 ± 27.2	183.9 ± 45.1	274.8 ± 56.4	437.6 ± 75.9	270.8 ± 81.3	432.2 ± 125.1
Cr	59.9 ± 43.1	61.2 ± 49.9	250.7 ± 141.0	93.1 ± 37.8	169.8 ± 4.7	164.0 ± 79.1
Hg	29.0 ± 8.3	29.3 ± 7.8	36.2 ± 5.2	33.5 ± 10.0	27.9 ± 1.3	38.8 ± 17.4
Ni	308.1 ± 162.7	676.2 ± 361.8	932.2 ± 193.8	378.9 ± 201.4	713.8 ± 203.3	412.4 ± 140.1

Reference dose (RfD) (µg/kg/day): As (0.3); Cd (1); Cr (3); Cu (40); Hg (0.1); Ni (20); Zn (300) (USEPA, 2005); Pb (3.5) (Djedjibegovic et al. 2020). Cancer slope factors (mg/kg/day): As (1.5); Cd (0.001); Cr (0.5); Ni (0.84) (USEPA, 2000; IRIS, 2017); Pb (0.00357) (Bacigalupo and Hale 2012)

## Discussion

*Cherax quadricarinatus* was introduced into Eswatini in the late 1980s on the banks of the Sand River Dam for aquaculture purposes and spread to South African rivers following flood events (Nunes et al. 2017a). The first reports of established wild populations of *C. quadricarinatus* were in 2002 (Komati River), 2012 (uSuthu River) and 2016 (Crocodile River) (Madzivanzira et al. 2020) and it is estimated to be spreading by approximately 16 km per year (Nunes et al. 2017b). By early 2024, *C. quadricarinatus* had spread to other river systems in the Kruger National Park (KNP), South Africa’s largest wildlife conservation area (Coetsee et al. 2024). The spread of this invasive crayfish not only poses ecological impacts – by altering river morphology, consuming and degrading macrophytes, invertebrates and fish communities – but also socio-economic impacts estimated to cause losses in fisheries of up to US$512 352 per year in e.g. Lake Kariba, Zimbabwe (Chakandinakira et al. 2023). However, with an increase in established wild populations of *C. quadricarinatus*, several local low-income communities are increasingly relying on these crayfish as an inexpensive food source (Nunes et al. 2017b).

### Element Bioaccumulation in *Cherax quadricarinatus*

Limited studies are available on element levels in *C. quadricarinatus* in natural settings. One study was conducted on crayfish from an urban impacted reservoir in Central Java, Indonesia (Rahayu et al. 2020), and in the Kafue River Basin, Zambia that drains intensive mining areas (Nakayama et al. 2010). The study in Indonesia only assessed concentrations of Cd, Cu, Pb and Zn, while the study in the Kafue River Basin assessed concentrations of Cd, Cr, Cu, Ni, Pb and Zn, with no data on As and Hg in *C. quadricarinatus*. The element accumulation order in *C. quadricarinatus* from the PRF was Zn > Cu > Pb > Cr > As > Ni > Hg > Cd. This was similar to both other studies, except Cr accumulation was higher than Pb in the Kafue River, while Pb accumulation was the highest in Central Java (Nakayama et al. 2010; Rahayu et al. 2020). The concentrations recorded in the PRF are generally in the same range as reported in the Kafue River Basin, except for higher Pb concentrations in the Phongolo River, while Cd concentrations in the PRF were approximately a factor of 10 higher compared to the concentrations in the Kafue River Basin. This indicates that even in a conservation area such as the NGR, aquatic biota can accumulate high pollutant concentrations that originate from land-use activities outside the conservation area, as was also the case reported for organochlorines in fish (Volschenk et al. 2019), elements in water and sediments (van Rooyen et al. 2022) and Hg in macroinvertebrates and fish (van Rooyen et al. 2023). The Hg concentrations measured in *C. quadricarinatus* during the present study were higher compared to the Hg concentrations in redbreast tilapia (*Coptodon rendalli*), Mozambique tilapia (*Oreochromis mossambicus*), and brown squeaker (*Synodontis zambezensis*), while lower compared to sharptooth catfish (*Clarias gariepinus*), silver catfish (*Schilbe intermedius*) and tiger fish (*Hydrocynus vittatus*) that have been collected in the PRF (van Rooyen et al. 2023).

### Human Health Risks

Since the present study is the first to report human health risks associated with the consumption of *C. quadricarinatus*, the risks cannot be compared to previous studies. However, there are several studies that assessed the human health risks associated with the consumption of mainly three fish species (common carp - *Cyprinus carpio*, Mozambique tilapia – *O. mossambicus*, and sharptooth catfish – *C. gariepinus*) in South Africa (Addo-Bediako et al. 2014; Jooste et al. 2015; Erasmus et al. 2022b, 2024; Stevens et al. 2023). The non-carcinogenic risks associated with As in *C. quadricarinatus* were lower compared to all three fish species collected from the Hex River, impacted by platinum mining activities (Erasmus et al. 2022b), as well as *C. gariepinus* collected from the Orange-Vaal River Basin, impacted by gold mining, industrial and urban activities (Erasmus et al. 2024). However, these HQ values for As in the PRF were twice as high compared to *C. gariepinus* and *O. mossambicus* collected from the Olifants River that is impacted by mining, industrial and urban activities (Addo-Bediako et al. 2014; Jooste et al. 2015). Only two other studies have assessed non-carcinogenic risks for Hg in South Africa, and the HQ values for Hg in the PRF were in the same range as for *C. gariepinus* collected from platinum and gold mining impacted rivers (Stevens et al. 2023), while lower compared to *C. gariepinus* collected from gold mining impacted rivers in the Orange-Vaal River Basin (Erasmus et al. 2024). The only studies that have assessed carcinogenic risks associated with the consumption of these three fish species in South Africa include Erasmus et al. (2022b), 2024), in the Hex River and the Orange-Vaal River Basin, respectively. The cancer risks associated with As in *C. quadricarinatus* collected from the PRF were lower, while cancer risks associated with Cr and Ni were in the same range compared to both studies. According to Coetzee et al. (2015) the most common aquatic species consumed in the PRF are *C. rendalli* > *O. mossambicus* > *S. zambezensis* > *C. gariepinus* > *S. intermedius* > *H. vittatus*. Considering that Hg levels in the crayfish were higher compared to the lower trophic fish species (tilapia species), while higher than those in the predator species (catfish and tiger fish), data from this study indicate that local consumers would be at a higher risk if they replaced their diet of tilapia species with the invasive crayfish as protein source.

## Conclusions

The present study assessed the levels of eight potentially toxic elements in *C. quadricarinatus* from the PRF. The elevated concentrations of As and Hg, as well as As, Cr and Ni in these crayfish have the potential to pose non-carcinogenic and carcinogenic health risks to human consumers, respectively. This can have larger implications if local communities change their diet to prefer the crayfish over tilapia species, as this can pose a higher exposure risk, while preference should rather be given to consume the crayfish above catfish and tiger fish as this can lower the exposure risk. Due to the mobility of elements, especially Hg, elevated concentrations can even be found in protected or conservation areas as they are normally downstream of several anthropogenic sources such as agricultural, industrial, mining and urban activities. This study highlights the need for monitoring programs, not only to assess the ecological and invasive status and distribution of these crayfish but also the element levels in them.

## Electronic supplementary material

Below is the link to the electronic supplementary material.

Supplementary Material 1

## References

[CR1] Acosta AA, Netherlands EC, Retief F, de Necker L, du Preez L, Truter M, Alberts R, Gerber R, Wepener V, Malherbe W, Smit NJ (2021) Conserving freshwater biodiversity in an African subtropical wetland: South Africa’s lower Phongolo River and floodplain. In: Kideghesho J (ed) Managing Wildlife in a changing World. IntechOpen, London

[CR2] Addo-Bediako A, Marr SM, Jooste A, Luus-Powell WJ (2014) Are metals in the muscle tissue of Mozambique tilapia a threat to human health? A case study of two impoundments in the Olifants River, Limpopo province, South Africa. Ann Limnol – Int J Lim 50:201–210. 10.1051/limn/2014091

[CR3] Bacigalupo C, Hale B (2012) Human health risks of pb and as exposure via consumption of home garden vegetables and incidental soil and dust ingestion: a probabilistic screening tool. Sci Total Environ 423:27–38. 10.1016/j.scitotenv.2012.01.057 22386683 10.1016/j.scitotenv.2012.01.057

[CR4] Chakandinakira AT, Madzivanzira TC, Mashonga S, Muzvondiwa JV, Ndlovu N, South J (2023) Socioeconomic impacts of Australian redclaw crayfish *Cherax quadricarinatus* in Lake Kariba. Biol Invasions 25:2801–2812. 10.1007/s10530-023-03074-8

[CR5] Coetsee C, Smit I, Kruger N, Spear D, Freitag-Ronaldson S (2024) South African National Parks 22/23 Research Report. SANParks, Cape Town

[CR6] Coetzee HC, Nell W, van Eeden ES, de Crom EP (2015) Artisanal fisheries in the Ndumo area of the lower Phongolo River Floodplain, South Africa. Koedoe 57:1248. 10.4102/koedoe.v57i1.1248

[CR7] de Moor I (2002) Potential impacts of alien freshwater crayfish in South Africa. Afr J Aquat Sci 27:125–139. 10.2989/16085914.2002.9626584

[CR8] de Necker L, Neswiswi T, Greenfield R, van Vuren J, Brendonck L, Wepener V, Smit N (2019) Long-term water quality patterns of a flow regulated tropical lowland river. Water 12:37. 10.3390/w12010037

[CR9] Djedjibegovic J, Marjanovic A, Tahirovic D, Caklovica K, Turalic A, Lugusic A, Omeragic E, Sober M, Caklovica F (2020) Heavy metals in commercial fish and seafood products and risk assessment in adult population in Bosnia and Herzegovina. Sci Rep 10:13238. 10.1038/s41598-020-70205-9 32764674 10.1038/s41598-020-70205-9 PMC7411038

[CR10] du Preez L, Smit NJ (2013) Double blow: alien crayfish infected with invasive temnocephalan in South African waters. S Afr J Sci 109:2013–0109. 10.1590/sajs.2013/20130109

[CR11] Dube T, de Necker L, van Vuren JHJ, Wepener V, Smit NJ, Brendonck L (2017) Spatial and temporal variation of invertebrate community structure in flood-controlled tropical floodplain wetlands. J Freshw Ecol 32:1–15. 10.1080/02705060.2016.1230562

[CR13] Erasmus JH, Malherbe W, Zimmermann S, Lorenz AW, Nachev M, Wepener V, Sures B, Smit NJ (2020) Metal accumulation in riverine macroinvertebrates from a platinum mining region. Sci Total Environ 703:134738. 10.1016/j.scitotenv.2019.134738 31731169 10.1016/j.scitotenv.2019.134738

[CR14] Erasmus JH, Smit NJ, Gerber R, Schaeffner BC, Nkabi N, Wepener V (2022a) Total mercury concentrations in sharks, skates and rays along the South African coast. Mar Pollut Bull 184:114142. 10.1016/j.marpolbul.2022.114142 36182787 10.1016/j.marpolbul.2022.114142

[CR16] Erasmus JH, Zimmermann S, Smit NJ, Malherbe W, Nachev M, Sures B, Wepener V (2022b) Human health risks associated with consumption of fish contaminated with trace elements from intensive mining activities in a peri-urban region. Sci Total Environ 825:154011. 10.1016/j.scitotenv.2022.154011 35192810 10.1016/j.scitotenv.2022.154011

[CR12] Erasmus JH, Herselman S, Wepener V (2023) Element concentrations in muscle and liver tissue of two eel species from the Incomati River, Mozambique. Bull Environ Contam Toxicol 111:34. 10.1007/s00128-023-03795-5 37690070 10.1007/s00128-023-03795-5 PMC10493202

[CR15] Erasmus JH, Truter M, Smit NJ, Nachev M, Sures B, Wepener V (2024) Element contamination of the Orange-Vaal River basin, South Africa: a one health approach. Environ Sci Poll Res 31:29886–29901. 10.1007/s11356-024-32932-8 10.1007/s11356-024-32932-8 PMC11058963 38589590

[CR17] Heath RGM, du Preez HH, Genthe B, Avenant-Oldewage A (2004) Freshwater Fish and Human Health. Reference guide. WRC Report No. TT212/04. Water Research Commission, Pretoria

[CR18] Hendriks H, Rossouw JN (2009) Adopt-a-River Programme phase II: development of an implementation plan. Water Resource Quality Situation Assessment. Department of Water Affairs, Pretoria

[CR19] Integrated Risk Information System (IRIS) (2017) IRIS assessments of United States Environmental Protection Agency. https://iris.epa.gov/AtoZ/?list_type=alpha (accessed 22 May 2024)

[CR20] Jooste A, Marr SM, Addo-Bediako A, Luus-Powell WJ (2015) Sharptooth catfish shows its metal: a case study of metal contamination at two impoundments in the Olifants River, Limpopo River system, South Africa. Ecotoxicol Environ Saf 112:96–104. 10.1016/j.ecoenv.2014.10.033 25463859 10.1016/j.ecoenv.2014.10.033

[CR21] lnkomati-Usuthu Catchment Agency (IUCMA) (2014) Annual Water Quality Report for the Inkomati-Usuthu Water Management Area 2014/2015, the National Government of South Africa. https://nationalgovernment.co.za/entity_annual/660/2015-inkomatiusuthu-catchment-management-agency-annual-report.pdf (accessed 27 May 2024).

[CR24] Madzivanzira TC, South J, Wood LE, Nunes AL, Weyl OLF (2020) A review of freshwater crayfish introductions in Africa. Rev Fish Sci Aquac 29:218–241. 10.1080/23308249.2020.1802405

[CR23] Madzivanzira TC, South J, Weyl OLF (2021) Invasive crayfish outperform Potamonautid crabs at higher temperatures. Freshw Biol 66:978–991. 10.1111/fwb.13691

[CR25] Madzivanzira TC, Weyl OLF, South J (2022) Ecological and potential socioeconomic impacts of two globally invasive crayfish. NeoBiota 72:25–43. 10.3897/neobiota.72.71868

[CR22] Madzivanzira TC, Chakandinakira AT, Mungenge CP, O’Brien G, Dalu T, South J (2023) Get it before it gets to my catch: misdirection traps to mitigate against socioeconomic impacts associated with crayfish invasion. Manag Biol Invasion 14:335–346. 10.3391/mbi.2023.14.2.10

[CR26] Nakayama SMM, Ikenaka Y, Muzandu K, Choongo K, Oroszlany B, Teraoka H, Mizuno N, Ishizuka M (2010) Heavy metal accumulation in lake sediments, fish (*Oreochromis niloticus* and *Serranochromis thumbergi*), and crayfish (*Cherax quadricarinatus*) in Lake Itezhi-Tezhi and Lake Kariba, Zambia. Arch Environ Contam Toxicol 59:291–300. 10.1007/s00244-010-9483-8 20162262 10.1007/s00244-010-9483-8

[CR27] Nunes AL, Zengeya TA, Hoffman AC, Measey GJ, Weyl OLF (2017a) Distribution and establishment of the alien Australian redclaw crayfish, *Cherax quadricarinatus*. South Afr Swazil PeerJ 5:e3135. 10.7717/peerj.3135 10.7717/peerj.3135 PMC5399870 28439454

[CR28] Nunes AL, Zengeya TA, Measey GJ, Weyl OLF (2017b) Freshwater crayfish invasions in South Africa: past, present and potential future. Afr J Aquat Sci 42:309–323. 10.2989/16085914.2017.1405788

[CR29] Rahayu DRUS, Anggoro S, Soeprobowati TR (2020) Potential threat of heavy metal accumulation in aquatic biota from Wadaslintang Reservoir, Central Java, Indonesia. Technol Rep Kansai Univ 62:2675–2683

[CR31] South J, McCard M, Khosa D, Mofu L, Madzivanzira TC, Dick JTA, Weyl OLF (2019) The effect of prey identity and substrate type on the functional response of a globally invasive crayfish. NeoBiota 52:9–24. 10.3897/neobiota.52.39245

[CR30] South J, Madzivanzira TC, Tshali N, Measey J, Weyl OLF (2020) In a pinch: mechanisms behind potential biotic resistance toward two invasive crayfish by native African freshwater crabs. Front Ecol Evol 8:72. 10.3389/fevo.2020.00072

[CR32] Stevens EL, Wepener V, Erasmus JH (2023) Assessment of human health risks through the consumption of mercury contaminated fish from gold and platinum mining areas. Int J Environ Anal Chem. 10.1080/03067319.2023.2290217

[CR33] United States Environmental Protection Agency (USEPA) (2000) Guidance for Assessing Chemical Contaminant Data for use in Fish Advisories. Volume 2: Risk Assessment and Fish Consumption Limits. third ed. EPA 823-B-00–008. Office of Water, Washington, DC

[CR34] United States Environmental Protection Agency (USEPA) (2005) Guidelines for carcinogen risk assessment. https://www.epa.gov/sites/default/files/2013-09/documents/cancer_guidelines_final_3-25-05.pdf (accessed 22 May 2024)

[CR36] van Rooyen D, Gerber R, Smit NJ, Wepener V (2022) An assessment of water and sediment quality of aquatic ecosystems within South Africa’s largest floodplain. Afr J Aquat Sci 47:474–488. 10.2989/16085914.2022.2124946

[CR35] van Rooyen D, Erasmus JH, Gerber R, Nachev M, Sures B, Wepener V, Smit NJ (2023) Bioaccumulation and trophic transfer of total mercury through the aquatic food webs of an African sub-tropical wetland system. Sci Total Environ 889:164210. 10.1016/j.scitotenv.2023.164210 37196965 10.1016/j.scitotenv.2023.164210

[CR37] Vilane BRT, Tembe L (2016) Water quality assessment upstream of the Great Usuthu River in Swaziland. J Agric Sci Eng 2:57–65

[CR38] Volschenk CM, Gerber R, Mkhonto MT, Ikenaka Y, Yohannes YB, Nakayama S, Ishizuka M, van Vuren JHJ, Wepener V, Smit NJ (2019) Bioaccumulation of persistent organic pollutants and their trophic transfer through the food web: human health risks to the rural communities reliant on fish from South Africa’s largest floodplain. Sci Total Environ 685:1116–1126. 10.1016/j.scitotenv.2019.06.144 31390702 10.1016/j.scitotenv.2019.06.144

[CR39] Zengeya TA, Lombard RJ, Nelwamondo VE, Nunes AL, Measey GJ, Weyl OLF (2022) Trophic niche of an invasive generalist consumer: Australian redclaw crayfish, *Cherax quadricarinatus*, in the Inkomati River Basin, South Africa. Austral Ecol 47:1480–1494. 10.1111/aec.13230

