# Supplementary material for: Elements in Invasive Redclaw Crayfish Cherax quadricarinatus Pose Human Health Risks in the Largest Floodplain System of South Africa
Source: Bull Environ Contam Toxicol. 2024 Oct 11;113(4):48. doi: 10.1007/s00128-024-03963-1 (PMC11469965; doi:10.1007/s00128-024-03963-1)
Supplement: Supplementary file 1 — Supplementary Material 1 [file 128_2024_3963_MOESM1_ESM.docx]

**Elements in invasive redclaw crayfish *Cherax quadricarinatus* pose human health risks in the largest floodplain system of South Africa**

Johannes H. Erasmus*, Wynand Malherbe, Nico J. Smit, Victor Wepener

Water Research Group, Unit for Environmental Sciences and Management, North-West University, Potchefstroom 2520, South Africa

* Corresponding author: J.H. Erasmus (22119809@mynwu.ac.za)

Email address:

Johannes H. Erasmus 22119809@mynwu.ac.za

Wynand Malherbe wynand.malherbe@nwu.ac.za

Nico J. Smit nico.smit@nwu.ac.za

Victor Wepener victor.wepener@nwu.ac.za

ORCID ID:

Johannes H. Erasmus 0000-0001-9056-5424

Wynand Malherbe 0000-0003-1852-5641

Nico J Smit 0000-0001-7950-193X

Victor Wepener 0000-0002-9374-7191

Supplementary data

**Table S1:** Biometric data (number of individuals, total body length, and weight) of Cherax quadricarinatus collected from the Pongolo River Floodplain, South Africa.

| Basin | Locality | n | Total body length (mm) | Weight (g) |
| --- | --- | --- | --- | --- |
| Phongolo River | Site 1 | 3 | 145 ± 40.9 | 23.3 ± 11.6 |
|  | Site 2 | 7 | 134 ± 28.6 | 27.5 ± 16.1 |
|  | Site 3 | 3 | 162 ± 25.7 | 30.5 ± 0.81 |
| uSuthu River | Site 4 | 8 | 166 ± 38.2 | 56.2 ± 24.3 |
|  | Site 5 | 3 | 188 ± 71.8 | 73.3 ± 66.6 |
|  | Site 6 | 8 | 159 ± 32.4 | 45.0 ± 21.4 |


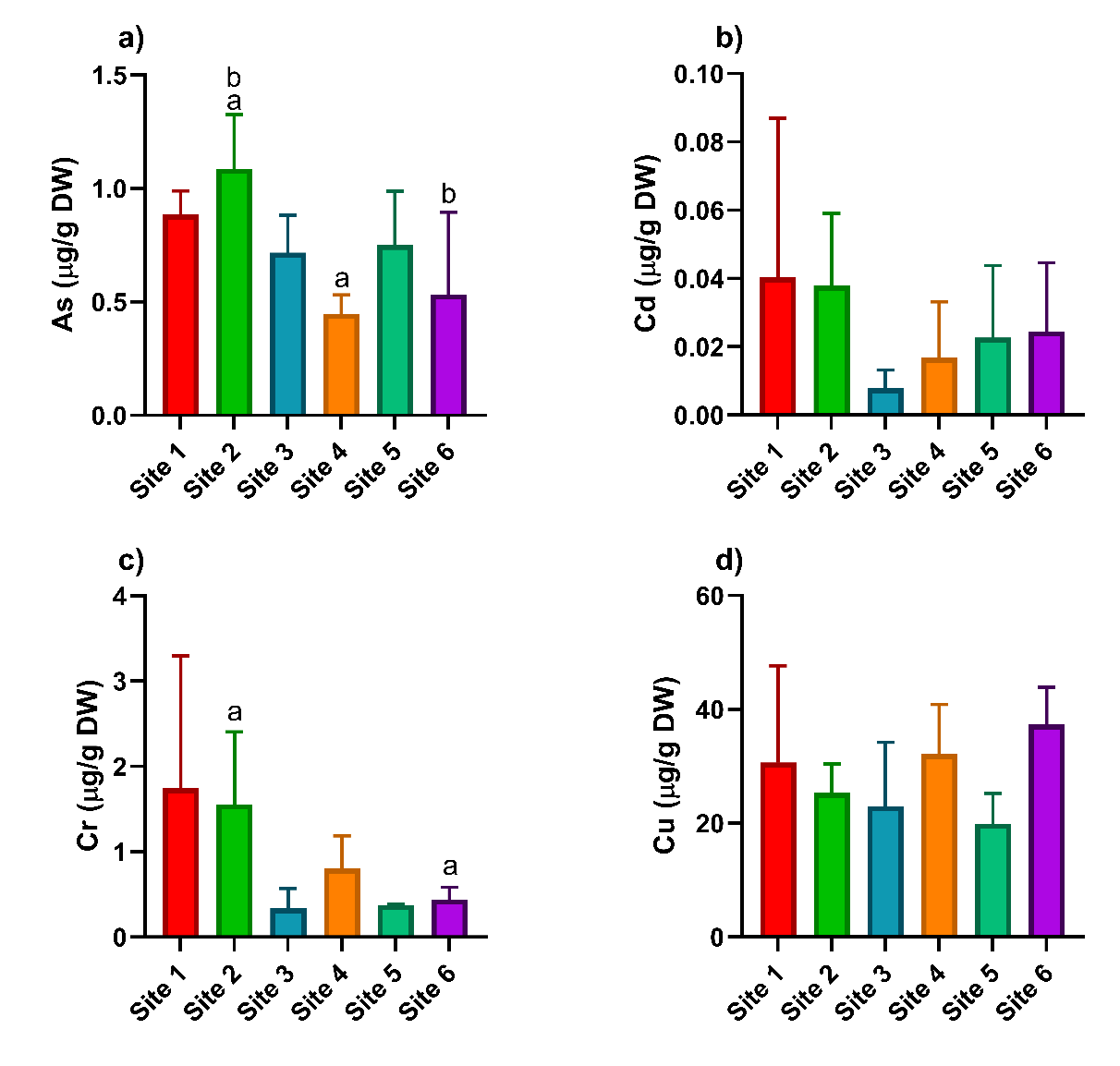


**Figure S1:** Mean concentrations (µg/g DW) of As (a), Cd (b), Cr (c) and Cu (d) with standard deviation of the mean in Cherax quadricarinatus tails collected from sites associated with the Phongolo River (Sites 1–3) and the uSuthu River (Sites 4–6), South Africa. Common alphabetical superscripts indicate significant differences between sites.


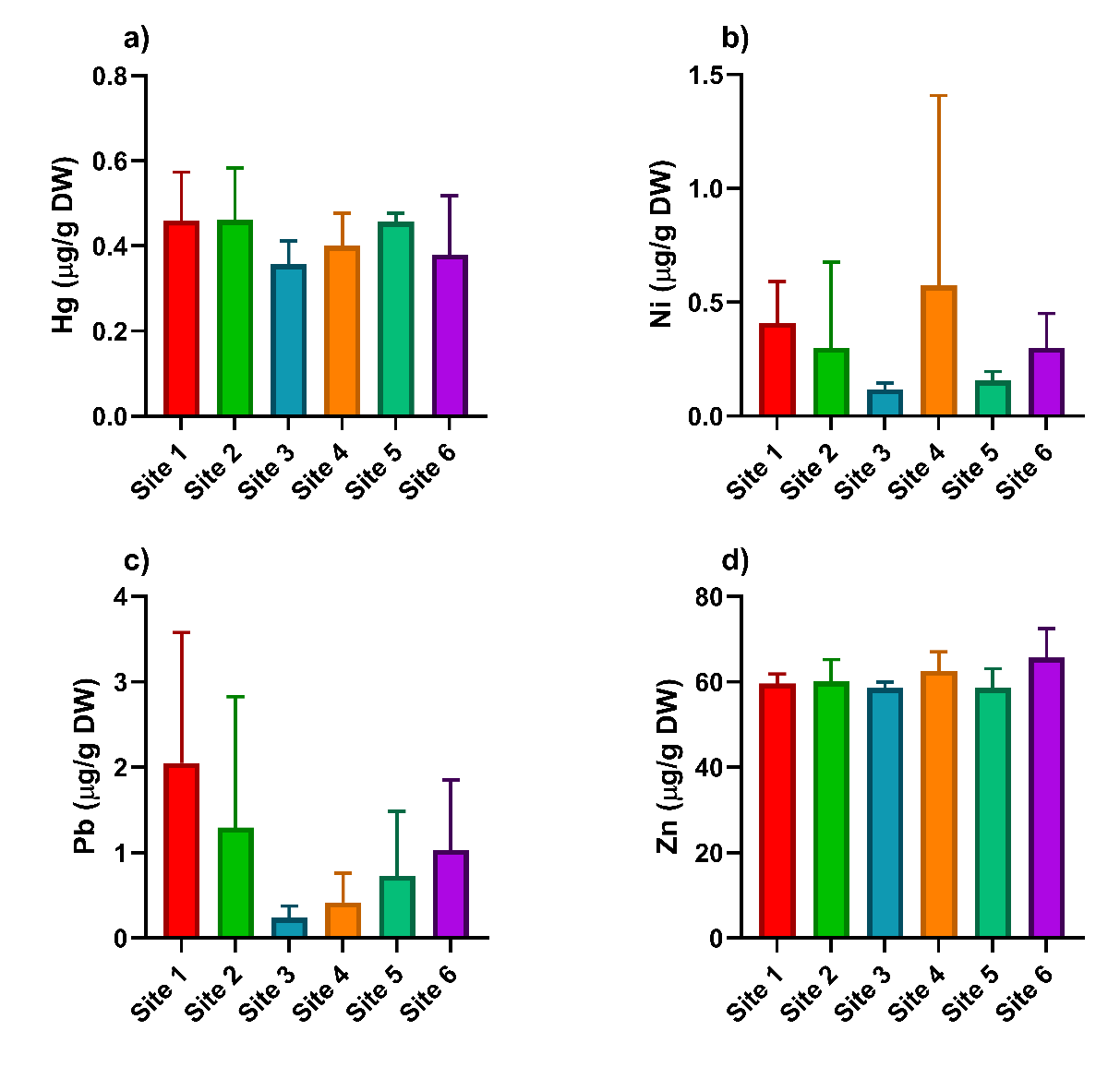


**Figure S2:** Mean concentrations (µg/g DW) of Hg (a), Ni (b), Pb (c) and Zn (d) with standard deviation of the mean in Cherax quadricarinatus tails collected from sites associated with the Phongolo River (Sites 1–3) and the uSuthu River (Sites 4–6), South Africa.

**Table S2:** The mean and standard deviation of hazard quotients (HQs) for non-carcinogenic risk and cancer risk (CR) for Cherax quadricarinatus collected from the Phongolo River Floodplain, South Africa. The values were calculated on the element concentration in crayfish tail tissue, supposing a person of 60 kg consumes one crayfish meal (150 g) twice a week for HQ or daily for CR. Hazard quotients of HQ > 1, indicating a high probability of adverse health effects, and CR > 10^-4^, indicating an unacceptable risk to humans who consume these crayfish are indicated in bold.

| Elements | Phongolo River | | | uSuthu River | | |
| --- | --- | --- | --- | --- | --- | --- |
|  | Site 1 | Site 2 | Site 3 | Site 4 | Site 5 | Site 6 |
| Hazard quotient for non-carcinogenic risk (HQ) | | | | | | |
| As | **1.0 ± 0.12** | **1.3 ± 0.27** | 0.82 ± 0.19 | 0.51 ± 0.10 | 0.86 ± 0.27 | 0.61 ± 0.42 |
| Cd | 0.014 ± 0.016 | 0.013 ± 0.007 | 0.003 ± 0.002 | 0.006 ± 0.006 | 0.008 ± 0.007 | 0.008 ± 0.007 |
| Cr | 0.20 ± 0.18 | 0.18 ± 0.10 | 0.039 ± 0.027 | 0.092 ± 0.043 | 0.043 ± 0.012 | 0.051 ± 0.016 |
| Cu | 0.26 ± 0.15 | 0.22 ± 0.043 | 0.20 ± 0.10 | 0.28 ± 0.075 | 0.17 ± 0.046 | 0.32 ± 0.056 |
| Hg | **1.6 ± 0.39** | **1.6 ± 0.42** | **1.2 ± 0.19** | **1.4 ± 0.27** | **1.6 ± 0.07** | **1.3 ± 0.48** |
| Ni | 0.007 ± 0.003 | 0.005 ± 0.007 | 0.002 ± 0.001 | 0.010 ± 0.014 | 0.003 ± 0.001 | 0.005 ± 0.003 |
| Pb | 0.20 ± 0.15 | 0.13 ± 0.15 | 0.023 ± 0.013 | 0.041 ± 0.033 | 0.072 ± 0.075 | 0.10 ± 0.08 |
| Zn | 0.068 ± 0.003 | 0.070 ± 0.006 | 0.067 ± 0.002 | 0.072 ± 0.005 | 0.067 ± 0.005 | 0.075 ± 0.008 |
| Carcinogenic risk (CR) (10^-4^) | | | | | | |
| As | **11.0 ± 1.3** | **13.5 ± 3.0** | **8.9 ± 2.1** | **5.6 ± 1.0** | **9.3 ± 2.9** | **6.6 ± 4.5** |
| Cd | 0.0003 ± 0.0004 | 0.0003 ± 0.0002 | 0.0001 ± 0.0001 | 0.0002 ± 0.0002 | 0.0002 ± 0.0002 | 0.0002 ± 0.0001 |
| Cr | **7.2 ± 6.4** | **6.4 ± 3.5** | **1.4 ± 0.97** | **3.3 ± 1.6** | **1.6 ± 0.04** | **1.8 ± 0.58** |
| Ni | **2.8 ± 1.3** | **2.1 ± 2.6** | 0.82 ± 0.19 | **4.0 ± 5.8** | **1.1 ± 0.27** | **2.1 ± 1.0** |
| Pb | 0.061 ± 0.045 | 0.038 ± 0.045 | 0.007 ± 0.004 | 0.012 ± 0.010 | 0.021 ± 0.022 | 0.031 ± 0.024 |

Reference dose (RfD) (µg/kg/day): As (0.3); Cd (1); Cr (3); Cu (40); Hg (0.1); Ni (20); Zn (300) (USEPA, 2005); Pb (3.5) (Djedjibegovic et al. 2020). Cancer slope factors (mg/kg/day): As (1.5); Cd (0.001); Cr (0.5); Ni (0.84) (USEPA, 2000; IRIS, 2017); Pb (0.00357) (Bacigalupo and Hale, 2012).
